# NpCIPK6–NpSnRK1 module facilitates intersubgeneric hybridization barriers in water lily (*Nymphaea*) by reducing abscisic acid content

**DOI:** 10.1093/hr/uhae289

**Published:** 2024-10-23

**Authors:** Ping Zhou, Jingwen Li, Huiyan Jiang, Zhijuan Yang, Chunqing Sun, Hongyan Wang, Qun Su, Qijiang Jin, Yanjie Wang, Yingchun Xu

**Affiliations:** Key Laboratory of Landscaping, Ministry of Agriculture and Rural Affairs, Key Laboratory of Biology of Ornamental Plants in East China, College of Horticulture, Nanjing Agricultural University, No. 666 Binjiang Avenue, Jiangbei New District, Nanjing, Jiangsu 210095, China; Sanya Research Institute of Nanjing Agricultural University, Building 9, Wutong Industrial Park, Zhenzhou Road, Yazhou District, Sanya 572000, China; Key Laboratory of Landscaping, Ministry of Agriculture and Rural Affairs, Key Laboratory of Biology of Ornamental Plants in East China, College of Horticulture, Nanjing Agricultural University, No. 666 Binjiang Avenue, Jiangbei New District, Nanjing, Jiangsu 210095, China; Sanya Research Institute of Nanjing Agricultural University, Building 9, Wutong Industrial Park, Zhenzhou Road, Yazhou District, Sanya 572000, China; Key Laboratory of Landscaping, Ministry of Agriculture and Rural Affairs, Key Laboratory of Biology of Ornamental Plants in East China, College of Horticulture, Nanjing Agricultural University, No. 666 Binjiang Avenue, Jiangbei New District, Nanjing, Jiangsu 210095, China; Sanya Research Institute of Nanjing Agricultural University, Building 9, Wutong Industrial Park, Zhenzhou Road, Yazhou District, Sanya 572000, China; College of Breeding and Multiplication, Hainan University (Sanya Institute of Breeding and Multiplication), Yazhou District Huanjin Road, Sanya, Hainan 570228, China; Key Laboratory of Landscaping, Ministry of Agriculture and Rural Affairs, Key Laboratory of Biology of Ornamental Plants in East China, College of Horticulture, Nanjing Agricultural University, No. 666 Binjiang Avenue, Jiangbei New District, Nanjing, Jiangsu 210095, China; Zhenjiang Institute of Agricultural Science in Jiangsu Hilly Areas, No. 1 Hongjing Road, Huayang Town, Jurong 212400, China; Flower Research Institute, Guangxi Academy of Agricultural Science, 174 Daxue East Road, Nanning 530007, China; Flower Research Institute, Guangxi Academy of Agricultural Science, 174 Daxue East Road, Nanning 530007, China; Key Laboratory of Landscaping, Ministry of Agriculture and Rural Affairs, Key Laboratory of Biology of Ornamental Plants in East China, College of Horticulture, Nanjing Agricultural University, No. 666 Binjiang Avenue, Jiangbei New District, Nanjing, Jiangsu 210095, China; Sanya Research Institute of Nanjing Agricultural University, Building 9, Wutong Industrial Park, Zhenzhou Road, Yazhou District, Sanya 572000, China; Key Laboratory of Landscaping, Ministry of Agriculture and Rural Affairs, Key Laboratory of Biology of Ornamental Plants in East China, College of Horticulture, Nanjing Agricultural University, No. 666 Binjiang Avenue, Jiangbei New District, Nanjing, Jiangsu 210095, China; Sanya Research Institute of Nanjing Agricultural University, Building 9, Wutong Industrial Park, Zhenzhou Road, Yazhou District, Sanya 572000, China; Key Laboratory of Landscaping, Ministry of Agriculture and Rural Affairs, Key Laboratory of Biology of Ornamental Plants in East China, College of Horticulture, Nanjing Agricultural University, No. 666 Binjiang Avenue, Jiangbei New District, Nanjing, Jiangsu 210095, China; Sanya Research Institute of Nanjing Agricultural University, Building 9, Wutong Industrial Park, Zhenzhou Road, Yazhou District, Sanya 572000, China

## Abstract

Prefertilization hybridization barriers are the main causes of intersubgeneric hybridization challenges in water lily. However, the mechanism underlying low compatibility between pollen and stigma of water lily remains unclear. This study demonstrates that CBL-interacting protein kinase 6 (CIPK6) responded to the signaling exchange between incompatible pollen and stigma through interactions with SNF1-related kinase 1 (SnRK1) and promotes the accumulation of SnRK1 protein. Activated SnRK1 interacted with 9-*cis*-epoxycarotenoid dioxygenase 2 (NCED2) to promote its degradation, thereby inhibiting abscisic acid (ABA) synthesis. A decrease in ABA content in the stigma impaired the ABA-mediated removal of reactive oxygen species (ROS), ultimately resulting in the rejection of the incompatible pollen by the stigma. Our results highlight the essential role of the NpCIPK6–NpSnRK1–NpNCED2 module in conferring intersubgeneric hybridization barriers in water lily by interfering with ABA synthesis and promoting ROS accumulation. This study offers valuable mechanistic insights into cellular signaling and reproductive barriers in water lily as well as across other biological contexts.

## Introduction

Water lily, a perennial aquatic plant belonging to the family Nymphaeaceae and the genus *Nymphaea*, is extensively cultivated due to their high ornamental value, medicinal properties, and their capacity to purify water [1]. The economic, ecological, and evolutionary significance of water lily is well recognized [2]. The global distribution of water lily includes >50 species, primarily located in tropical, subtropical, and temperate regions [3]. According to their geographical distribution, water lily can be divided into two ecological types: tropical and hardy [4]. The tropical water lily exhibits a diverse range of colors and is intolerant to cold in high-latitude areas. In contrast, the hardy water lily displays limited color variations but is cold-resistant [3]. Consequently, hybridizing hardy and tropical water lilies is of great significance as it can result in new cultivars that are both cold-resistant and rich in color, thereby expanding the existing water lily germplasm resources.

Many attempts to hybridize tropical and hardy water lilies have been made in the past 150 years [5]. However, it is difficult to obtain hybrid seeds due to hybridization barriers between tropical and hardy water lilies [1, 6, 7], which greatly affects the efficiency of cross-breeding among water lily subgenera. Studies indicated that prefertilization hybridization barriers are the primary cause of the low fruiting rate in water lily subgenera. These barriers primarily manifest as low pollen germination rates on the stigma and abnormal pollen tube growth in the style, with the tip bending or swelling, thus preventing the pollen tube from entering the ovule [1, 6, 7]. The most prevalent issue during intersubgenus hybridization of water lily is poor pollen germination on the stigma.

Abscisic acid (ABA), an important plant hormone that is distributed in multiple tissues and organs, plays crucial roles in plant growth, development [8], and reproduction [9–11]. For instance, ABA regulates pollen germination and pollen tube growth [11–14]. In *Arabidopsis*, low temperature promotes ABA accumulation in anthers, and ABA content is negatively correlated with anther development [15], i.e. elevated ABA levels have been reported to prevent anther development, thereby reducing male fertility [16–18]. Moreover, elevated levels of ABA in anthers result in the excessive accumulation of reactive oxygen species (ROS). The ROS accumulation leads to oxidative damage, disruption of tapetum programmed cell death (PCD), and a subsequent reduction in male fertility [19]. These findings corroborate the detrimental impact of ABA on the regulation of male reproductive development. However, the regulation of ABA during plant reproduction appears paradoxical and may be associated with the dynamic equilibrium between ABA content and accumulation in various parts of pollen or stigma [13, 14, 20]. For instance, ABA can regulate the growth of pollen tubes by altering the availability of carbohydrates in the style [8]. Furthermore, the exogenous application of ABA has been demonstrated to enhance maize pollen germination and tube growth [8, 9]. In summary, ABA emerges as a significant regulator in the modulation of pollen fertility and germination. However, how ABA affects pollen development and germination has rarely been reported.

**Figure 1:**
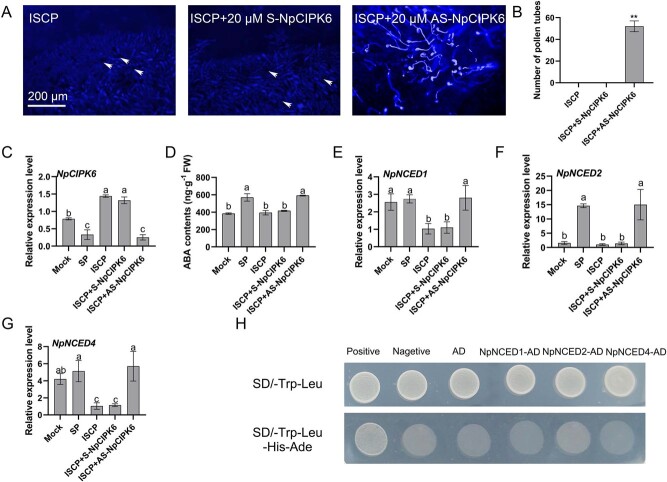
Silencing of *NpCIPK6* in ISCP stigma promotes pollen tube germination. (A) Aniline blue staining shows pollen growth in S-NpCIPK6- and AS-NpCIPK6-treated ISCP stigmas. Arrows point to ungerminated pollen grains. Scale bar = 200 μm. (B) Pollen germination in ISCP stigmas treated with S-NpCIPK6 and AS-NpCIPK6. ISCP stigmas served as the control. (C) Transcript level of *NpCIPK6* in S-NpCIPK6- and AS-NpCIPK6-treated ISCP heads detected by qRT-PCR analysis. (D) ABA content in ISCP columns treated with S-NpCIPK6 and AS-NpCIPK6. (E–G) qRT-PCR analysis of *NpNCED1*, *NpNCED2*, and *NpNCED4* in S-NpCIPK6- or AS-NpCIPK6-treated ISCP heads. (H) Yeast two-hybrid assay to verify the interactions between NpCIPK6 and three NpNCEDs. ISCP in the figure indicates intersubgenus cross-pollinated. The data presented are the mean values ± standard deviation (SD) from three independent replicates. Statistical analysis using a two-sided Student’s *t*-test revealed significant differences (*P* < 0.01) denoted by **. Different letters denote significant differences (*P* < 0.05, one-way ANOVA).

In our previous study, we observed elevated ABA levels in the stigma of water lilies subsequent to both self-pollination (SP) and cross-pollination (CP) within the same subgenus, in comparison to intersubgeneric hybridization (ISCP) [21]. we observed elevated ABA levels in the stigma of water lilies subsequent to both SP and CP within the same subgenus, in comparison to intersubgeneric hybridization (ISCP). Based on weighted coexpression analysis, *NpCIPK6* was found to be a candidate gene for regulating the barrier to interspecific hybridization in water lily [21].

In this study, we demonstrate the pivotal role of *NpCIPK6* in facilitating the establishment of barriers to intersubgeneric hybridization in water lilies. Specifically, the expression of *NpCIPK6* can be activated by the signaling effects of intersubgeneric hybridization, and NpCIPK6 can promote the kinase activity of NpSnRK1 by interacting with NpSnRK1. In addition, activated NpSnRK1 inhibited ABA synthesis in water lily stigmas by interacting with NpNCED2 and promote the protein degradation of NpNCED2. The consequent reduction in ABA levels within water lily stigmas disrupts the scavenging of ROS, thereby impeding the germination of incompatible pollen on water lily stigmas. This elucidation of the signaling cascade provides a conceptual framework for overcoming barriers to intersubgeneric hybridization in water lily and improving breeding efficiency.

## Results

### Sequence analysis and subcellular localization of NpCIPK6

In a preliminary study of ISCP treatment, we found that *NpCIPK6* was exclusively expressed in the stigma of water lily [21]. Therefore, we speculated that the high *NpCIPK6* expression contributes to the intersubgeneric hybridization barriers in water lily. To understand the role of NpCIPK6 in regulating inter-subgeneric hybridization, we constructed a phylogenetic tree of the *CIPK* gene family in water lily and other related species by means of protein sequence alignment (Fig. S2a). The water lily NpCIPK6 protein sequence showed high homology to that of *Glycine max*, *Oryza sativa*, and *Solanum lycopersicum*, and it formed a distinct branch with the AtCIPK6 of *Arabidopsis*. In addition, NpCIPK6 contains a catalytic kinase domain and a regulatory domain (Fig. S2b), and the activation loop of NpCIPK6 contains three conserved phosphorylation sites (threonine and serine). In the regulatory domain, a NAF/FTSL domain mediating the interaction between CIPK and CBL was identified (Fig. S2b), revealing that NpCIPK6 can receive Ca^2+^ signals. Subcellular localization analysis showed that the NpCIPK6-GFP fusion protein is expressed in the cell membrane and nucleus of *Nicotiana benthamiana* leaf (Fig. S2c).

### *NpCIPK6* knockdown promotes the germination of incompatible pollen on water lily stigma

To explore the functional role of NpCIPK6, we employed antisense oligonucleotides (AS-ODN) to downregulate *NpCIPK6* expression specifically in the stigma of water lily. Our findings revealed a significant augmentation in pollen germination on stigmas with AS-NpCIPK6 treatment, whereas the stigmas treated with S-NpCIPK6 showed no pollen germination (Fig. 1a, b). Subsequent quantitative real-time polymerase chain reaction (qRT-PCR) analysis substantiated the downregulation of *NpCIPK6* expression in the stigma with AS-NpCIPK6 treatment (Fig. 1c), thus indicating that the knockdown of *NpCIPK6* disrupts the intersubspecific hybridization barriers in water lily.

ABA in the stigma of water lily can regulate pollination affinity, and a preliminary study has shown a negative correlation between *NpCIPK6* expression trends and ABA content [21]. Our results indicated that AS-NpCIPK6 application significantly increased ABA content in the stigma of water lily (Fig. 1d). Further analysis revealed that *NpCIPK6* knockdown promotes the expression of three *NpNCED* genes, namely *NpNCED1*, *NpNCED2*, and *NpNCED4*, in the stigma (Fig. 1e–g). The results of yeast two-hybrid experiments showed that NpCIPK6 does not directly interact with these three NpNCEDs, suggesting that the regulation of NpNCED by NpCIPK6 may be indirect.

### NpCIPK6 interacts with NpSnRK1 to promote its kinase activity

To further understand the mechanism by which NpCIPK6 inhibits ABA synthesis, we performed a yeast two-hybrid screen of yeast libraries prepared from water lily stigma using NpCIPK6 as bait. Among the interacting proteins obtained from the screen, SNF1-related kinase 1 (SnRK1) sparked our interest. Phylogenetic analysis showed that NpSnRK1 clustered with the α subunit of SnRK1 from *Arabidopsis*, lotus, rice, and peach species (Fig. S3a). Further sequence alignment analysis revealed that NpSnRK1 from water lily shows very high similarity to the α subunit of SnRK1 from these species (Fig. S3b), indicating that NpSnRK1 belongs to the SNF1-related protein kinase catalytic subunit alpha (kin10) subfamily. Subcellular localization showed that the NpSnRK1-GFP fusion protein is expressed in the cell membrane and nucleus of *N. benthamiana* leaf (Fig. S3c).

**Figure 2:**
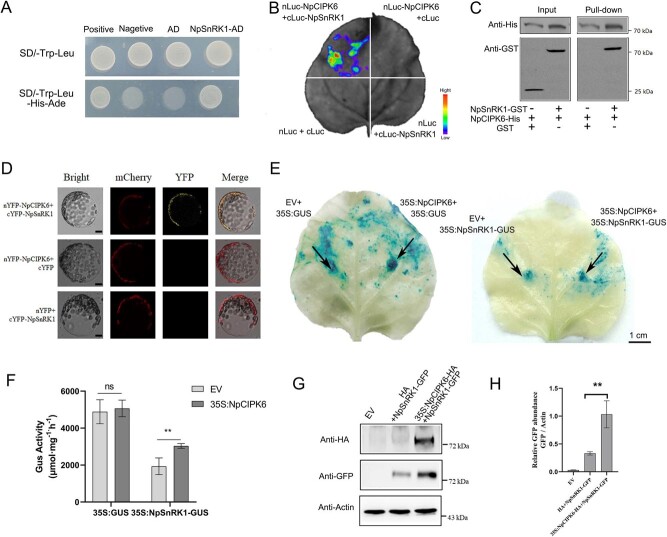
NpCIPK6 interacts with NpSnRK1 to promote its activity. (A) Yeast two-hybrid analysis of the interaction between NpCIPK6 and NpSnRK1. (B) Complementary luciferase imaging (LCI) assay of *NpCIPK6-nLuc* and *NpSnRK1-cLuc* in tobacco epidermal cells. Scale bar = 1 cm. (C) Pull-down analysis of NpSnRK1-GST with NpCIPK6-His. The GST tag served as the control. (D) BiFC assay of the interaction between nYFP-NpCIPK6 and cYFP-NpSnRK1 in tobacco protoplasts. Scale bar = 5 μm. (E, F) GUS activity analysis to investigate the mechanism underlying NpCIPK6–NpSnRK1 interactions. Scale bar = 1 cm. (G, H) Western blot assay of NpSnRK1 and NpCIPK6 using unconjugated GFP antibody. The data presented are the mean values ± SD from three independent replicates. Statistical significance was denoted as * for *P* < 0.05, ** for *P* < 0.01 and ns indicates no significant difference. Using a two-sided Student’s *t*-test.

Further validation using a yeast two-hybrid assay confirmed that NpCIPK6 directly interacts with NpSnRK1 (Fig. 2a). We transfected the *nLUC-NpCIPK6/cLUC-NpSnRK1* fusion vectors into the leaves of *N. benthamiana* for the luciferase complementation assay. Fluorescence signals were detected in regions of *nLUC-NpCIPK6* and *cLUC-NpSnRK1* coexpression but not in regions of *nLUC-NpCIPK6* and *cLUC*, *nLUC* and *NpSnRK1-cLUC*, or *nLUC* and *cLUC* coexpression. These findings further support the reciprocal relationship between NpCIPK6 and NpSnRK1 (Fig. 2b). In addition, the results of the pull-down assay confirmed the interaction between NpCIPK6 and NpSnRK1 *in vitro* (Fig. 2c). The bimolecular fluorescence complementation (BiFC) assay also revealed a strong yellow fluorescent protein signal at the cell membrane caused by the interaction of NpCIPK6 and NpSnRK1 (Fig. 2d).

To understand the regulatory role of NpCIPK6 on NpSnRK1, the fusion vectors *pSak277-35S: NpCIPK6* and *35S: NpSnRK1-GUS*, as well as *pYLZa06-35S: NpCIPK6-HA* and *35S: NpSnRK1-GFP*, were individually co-infiltrated into *N. benthamiana* leaves by *Agrobacterium*-mediated transformation. The results of the GUS staining assay revealed that the expression of *35S: NpSnRK1-GUS* yielded a light blue color, whereas the coexpression of *pSak277-35S: NpCIPK6* and *35S: NpSnRK1-GUS* yielded a dark blue color (Fig. 2e), suggesting that NpCIPK6 can promote NpSnRK1 activity. These findings further support this notion, as we observed a significant enhancement of GUS protein activity with co-overexpression of *pSak277-35S: NpCIPK6* and *35S: NpSnRK1-GUS* compared to overexpression of *35S: NpSnRK1-GUS* (Fig. 2f). The regulation of NpSnRK1 protein by NpCIPK6 was further verified by western blotting using a GFP antibody. The abundance of GFP protein was higher in *N. benthamiana* leaves that coexpressed *pYLZa06-35S: NpCIPK6-HA* and *35S: NpSnRK1-GFP* than in those that expressed *35S: NpSnRK1-GFP* alone (Fig. 2g, h), indicating that NpCIPK6 can interact with NpSnRK1 and promote NpSnRK1 protein accumulation.

### *NpSnRK1* knockdown promotes the germination of incompatible pollen on water lily stigma

The qRT-PCR was used to investigate the expression levels of *NpSnRK1* in the stigma under different treatments, including unpollinated (Mock), SP, CP, and ISCP. Our findings revealed a significant increase in *NpSnRK1* expression in ISCP-treated stigmas compared to those with SP and CP stigmas, consistent with the observed results for NpCIPK6 (Fig. S4a). Furthermore, a positive correlation was observed between *NpSnRK1* expression and its kinase activity in the stigma (Fig. S4b). These results suggested that NpSnRK1 likely plays a crucial role in regulating intersubgeneric hybridization barriers in water lily.

**Figure 3:**
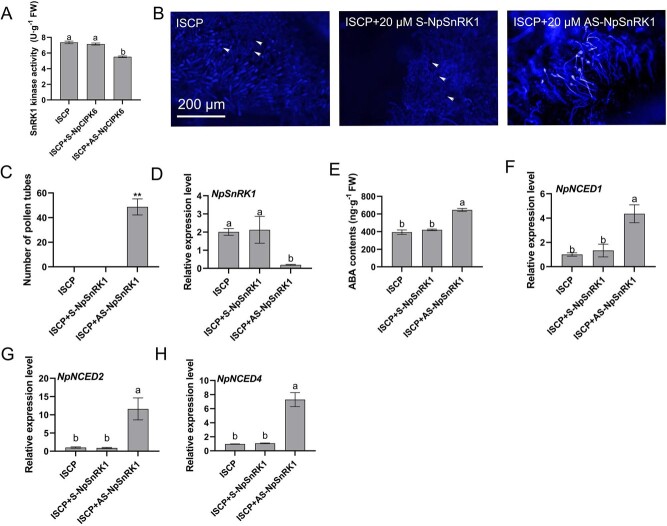
Silencing of *NpSnRK1* in ISCP stigmas promotes pollen tube germination. (A) Kinase activity assessment of NpSnRK1 in S-NpCIPK6- and AS-NpCIPK6-treated ISCP heads. (B) Aniline blue staining for observation of pollen growth in S-NpCIPK6- and AS-NpSnRK1-treated ISCP stigmas. Arrows point to ungerminated pollen grains. Scale bar = 200 μm. (C) Pollen germination on ISCP stigmas treated with S-NpSnRK1 and AS-NpSnRK1. (D) qRT-PCR analysis of *NpSnRK1* in S-NpSnRK1- and AS-NpSnRK1-treated ISCP heads. (E) Determination of ABA content in ISCP columns treated with S-NpSnRK1 and AS-NpSnRK1. (F–H) Relative transcript levels of *NpNCED1*, *NpNCED2*, and *NpNCED4* in S-NpSnRK1- and AS-NpSnRK1-treated ISCP heads detected by qRT-PCR. ISCP in the figure indicates intersubgenus cross-pollinated. The data presented are the mean values ± SD from three independent replicates. Statistical analysis using a two-sided Student’s *t*-test revealed significant differences (*P* < 0.01) denoted by **. Different letters denote significant differences (*P* < 0.05, one-way ANOVA).

To elucidate the epistatic relationship between NpCIPK6 and NpSnRK1, the stigma was treated with AS-NpCIPK6. The results showed that *NpCIPK6* knockdown decreased NpSnRK1 kinase activity in the stigma (Fig. 3a), suggesting that NpSnRK1 is a downstream target of NpCIPK6. These findings are consistent with the those from the GUS activity assays and western blot assays (Fig. 2e–h). To investigate the role of NpSnRK1 in regulating the formation of intersubgeneric hybridization barriers in water lilies, we employed antisense oligonucleotides to temporarily suppress *NpSnRK1* expression in stigmas of water lily (*Nymphaea* ‘Peter Slocum’). The results of qRT-PCR confirmed the reduced *NpSnRK1* expression in AS-NpSnRK1-treated stigma (Fig. 3d). A large number of pollen grains (from (*Nymphaea* ‘NangKwaug Fah’)) were found to germinate on the stigma treated with AS-NpSnRK1, whereas no pollen germination was observed on stigma treated with S-NpSnRK1 (Fig. 3b, c). The ABA content increased by 1.9-fold in AS-NpSnRK1-treated stigma compared with ISCP- and S-NpSnRK1-treated stigma (Fig. 3e). The results of qRT-PCR showed that the expression of *NpNCED1*, *NpNCED2*, and *NpNCED4*, which are involved in ABA biosynthesis, in stigma increased significantly upon AS-NpSnRK1 treatment (Fig. 3f–h), revealing that the NpCIPK6–NpSnRK1 module likely participates in the regulation of the intersubgeneric hybridization barriers by inhibiting ABA biosynthesis.

### NpSnRK1 interacts with NpNCED2 to induce its degradation

**Figure 4:**
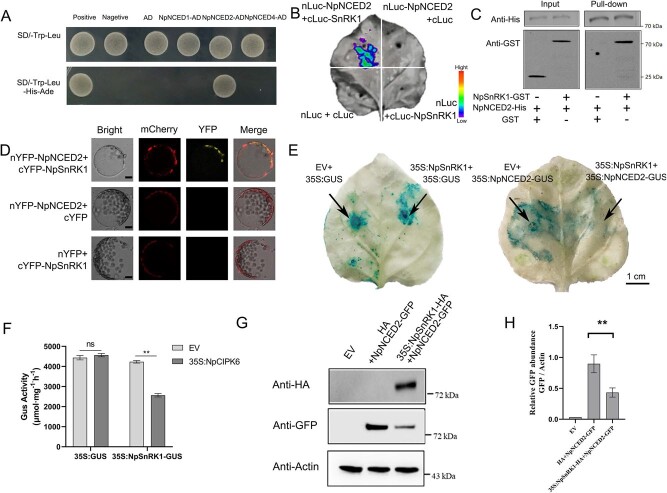
NpSnRK1 interacts with the NpNCED2 protein to induce its degradation. (A) Yeast two-hybrid analysis of the interaction between NpSnRK1 and NpNCED2. (B) Complementary LCI assay of *NpNCED2-nLuc* and *NpSnRK1-cLuc* in tobacco epidermal cells. Scale bar = 1 cm. (C) Pull-down analysis of NpSnRK1-GST with NpNCED2-His. The GST tag was used as the control. (D) BiFC assay of the interaction between *nYFP-NpNCED2* and *cYFP-NpSnRK1* in tobacco protoplasts. Scale bar = 5 μm. (E, F) GUS activity analysis for investigating the mechanism underlying NpSnRK1–NpNCED2 interactions. Scale bar = 1 cm. (G, H) Western blot analysis of NpSnRK1 on NpNCED2 using unconjugated GFP antibody. The data presented are the mean values ± SD from three independent replicates. Statistical significance was denoted as * for *P* < 0.05, ** for *P* < 0.01 and ns indicates no significant difference. Using a two-sided Student’s *t*-test.

To further explore the mechanism by which NpSnRK1 affects the expression of *NpNCED1*, *NpNCED2*, and *NpNCED4*, we investigated the interactions among NpSnRK1 and the three NpNCED proteins using the yeast two-hybrid assay. Direct interactions were detected between NpSnRK1 and NpNCED2 (Fig. 4a), consistent with the results of the pull-down assay (Fig. 4b). To investigate whether NpSnRK1 and NpNCED2 also interact *in vivo*, transient expression assays were conducted in leaves of *N. benthamiana*. The results revealed that that a fluorescent protein signal was produced upon the coexpression of *nLUC-NpNCED2* and *cLUC-NpSnRK1*. Conversely, no fluorescent signal was observed in *N. benthamiana* leaves expressing three combinations: *nLUC-NpNCED2* and *cLUC*, *cLUC-NpNCED2* and *nLUC*, and *nLUC* and *cLUC* (Fig. 4c). The results of the BiFC assay also revealed interaction between NpSnRK1 with NpNCED2 at the cell membrane (Fig. 4d).

*35S: NpNCED2-GUS* and *pSak277-35S: NpSnRK1*, as well as *35S: NpNCED2-GFP* and *pYLZa06-35S: NpSnRK1-HA*, were overexpressed in *N. benthamiana* leaves using *Agrobacterium*-mediated transformation. The results of the GUS staining and activity assay detected robust GUS staining in *N. benthamiana* leaves that overexpressed *35S: NpNCED2-GUS* alone and weak GUS staining in *N. benthamiana* leaves that overexpressed both *35S: NpNCED2-GUS* and *pSak277-35S: NpSnRK1* (Fig. 4e, f). Western blot experiments using GFP as the primary antibody detected a high GFP abundance in *N. benthamiana* leaves that overexpressed *35S:NpNCED2-GFP* alone but a low GFP abundance in *N. benthamiana* leaves that overexpressed both *35S: NpNCED2-GFP* and *pYLZa06-35S: NpSnRK1-HA* (Fig. 4g, h).

### Knockdown of *NpCIPK6* and *NpSnRK1* reduces ROS accumulation in water lily stigma

Excessive ROS accumulation in the stigma has been reported as an important mechanism of intersubgeneric hybridization barriers in water lily [7, 22]. In order to prove this, we applied an exogenous ROS scrubber (N, N′-Dimethylthiourea (DMTU), Sodium salicylate, Sodium pyruvate, and N-acetylcysteine (NAC)) to the stigmas of ISCP. We have found that DMTU, Sodium salicylate, Sodium pyruvate, and NAC could remove the content of ROS in the stigmas of water lily, and promote the germination of incompatibility pollen on the stigmas of water lily, further proving that high ROS content is the key to the formation of intergenus hybridization barriers of water lily (Fig. S5).

**Figure 5:**
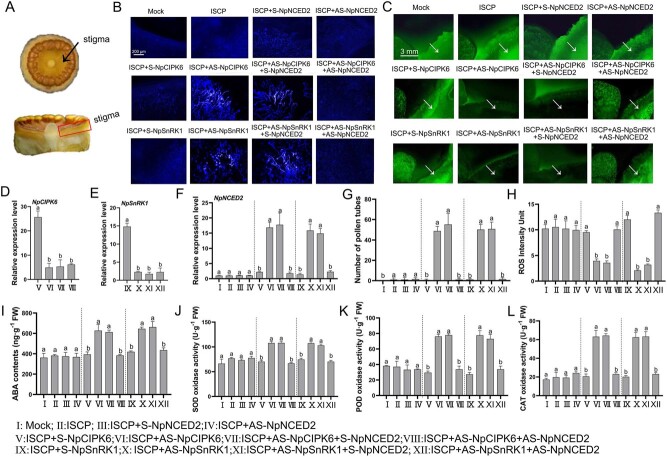
ROS accumulation and antioxidant enzyme activity in the stigmas of water lily. (A) Diagram of the stigma of water lily. The arrow shows the top view of the water lily stigma. The box shows the longitudinal cut of the water lily stigma. (B) The growth of intersubgeneric hybrid pollen in ISCP water lily stigmas under different S- or AS- treatments. (C) The ROS content in stigmas. (D–F) qRT-PCR analysis of *NpCIPK6*, *NpSnRK1*, and *NpNCED2* in ISCP water lily stigmas under different S- or AS- treatments. (G) The number of pollen tubes germinating on stigma of ISCP under different S- or AS- treatments. (H) Fluorescence index of ROS in stigmas of ISCP under different S-or AS-treatments. (I) Determination of ABA content in ISCP water lily stigmas under different S- or AS- treatments. (J–L) Activities of SOD, POD, and CAT in ISCP water lily stigmas under different S- or AS- treatments. The Mock in the figure represents an unpollinated stigma. ISCP in the figure indicates intersubgenus cross-pollinated. The data presented are the mean values ± SD from three independent replicates. Different letters denote significant differences (*P* < 0.05, one-way ANOVA).

To investigate whether NpCIPK6 and NpSnRK1 can affect intersubgeneric hybridization barriers through the regulation of ROS levels, we examined the ROS content, as well as the activities of antioxidant enzymes, in the stigma of water lily after AS-NpCIPK6 and AS-NpSnRK1 treatments. qRT-PCR analysis confirmed that AS-NpCIPK6, AS-NpSnRK1, and AS-NpNCED2 treatments could inhibit the expression of *NpCIPK6*, *NpSnRK1*, and *NpNCED2* in stigma of ISCP (Fig. 5d–f). Further studies showed that the application of AS-NpCIPK6 and AS-NpSnRK1 reduced ROS levels in stigmas treated with ISCP (Fig. 5c, h). Interestingly, after further treatment with AS-NpNCED2 in the AS-NpCIPK6- or AS-NpSnRK1-treated stigmas, the scavenging effect of AS-NpCIPK6 and AS-NpSnRK1 on ROS on the stigmas was inhibited, and the ROS content was significantly increased (Fig. 5c, h). Stigma pollen germination was negatively correlated with ROS content; the stigma with higher ROS content had lower pollen germination number (Fig. 5b, g). In addition, we also found that the ABA content in the stigmas was increased under AS-NpCIPK6 and AS-NpSnRK1 treatment, while the ABA content was significantly downregulated after the addition of AS-NpNCED2 (Fig. 5i). It was suggested that the increase in ABA under AS-NpCIPK6 and AS-NpSnRK1 treatment may be accomplished by promoting the expression of *NpNCED2* (Fig. 5i).

Previous studies have demonstrated the significant involvement of ABA in regulating the levels of ROS. Consistent with our previous findings, we have substantiated that ABA present in the water lily stigma enhances the activities of key antioxidant enzymes such as superoxide dismutase (SOD), peroxidase (POD), and catalase (CAT). Additionally, our studies have highlighted the crucial role of NpNCED2 in ABA synthesis within the water lily stigma [21]. These antioxidant enzymes, namely SOD, POD, and CAT, are vital in effectively scavenging ROS. Likewise, it has been reported that SOD, POD, and CAT in stigmas are essential for maintaining ROS homeostasis, thereby ensuring successful pollination processes [23, 24]. In this study, we found that the activities of SOD, POD, and CAT increased by 1.6-, 1.7-, and 2-fold in AS-NpCIPK6-treated stigma compared with S-NpCIPK6-treated stigma (Fig. 5j, k, l). Moreover, the effects of AS-NpCIPK6 on SOD, POD, and CAT activities depended on the regulation of *NpNCED2* (Fig. 5j, k, l). AS-NpSnRK1 treatment also obtained similar results, i.e. AS-NpSnRK1 treatment can increase the activities of SOD, POD, and CAT, and this phenomenon can be inhibited by AS-NpNCED2 treatment (Fig. 5j, k, l). These findings suggested that the NpCIPK6–NpSnRK1 module possibly inhibits the ABA-mediated antioxidant system by suppressing ABA biosynthesis, thereby preventing the scavenging of ROS from the stigma to inhibit the germination of incompatible pollen.

### Overexpression of *NpCIPK6* and *NpSnRK1* inhibited the germination of tobacco pollen on stigma

**Figure 6:**
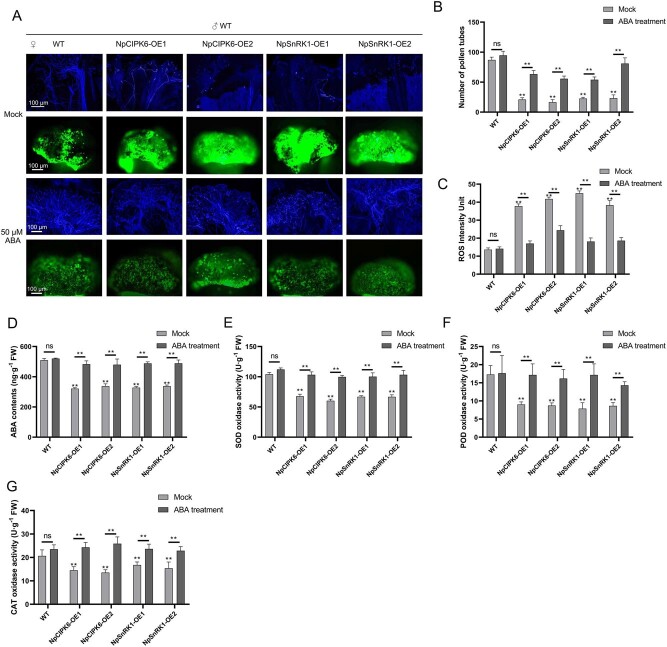
Overexpression of *NpCIPK6* and *NpSnRK1* inhibited the germination of tobacco pollen on stigma. (A) The germination of pollen on stigma and the accumulation of ROS in stigma of tobacco. Scale bar = 100 μm. (B) The number of germination pollen tubes on the stigma of tobacco. (C) Fluorescence intensity of ROS in tobacco stigma. (D) Determination of ABA content in tobacco stigma. (E–G) Activities of SOD, POD, and CAT in tobacco stigmas. The data presented are the mean values ± SD from three independent replicates. Statistical analysis using a two-sided Student’s *t*-test revealed significant differences (*P* < 0.01) denoted by **, ns indicates no significant difference.

To further verify the results that NpCIPK6 and NpSnRK1 regulate pollen germination on stigma, we obtained transgenic tobacco (*Nicotiana tabacum* L.) plants with overexpression of *NpCIPK6* and *NpSnRK1* by heterologous expression (Fig. S6). Wild-type tobacco (WT) was used as the paternal parent. The WT and the transgenic tobacco plants hosting *NpCIPK6* and *NpSnRK1* were used as the maternal parent for pollination. Pollen tube germination and ROS content in stigma were observed after pollination 3 h. The results showed that pollen germination was normal on stigma of WT, while the number of pollen germination on tobacco stigma overexpressing *NpCIPK6* and *NpSnRK1* was significantly decreased (Fig. 6a, b). Compared with stigma of WT, the ROS content in tobacco stigma overexpressing *NpCIPK6* and *NpSnRK1* maintained a higher level (Fig. 6a, c). In addition, ABA content in transgenic tobacco stigma was significantly downregulated compared to WT (Fig. 6 d). The activity of antioxidant enzymes was positively correlated with ABA content (Fig. 6 e, f, g). To further understand the relationship between ROS and ABA content, ABA was applied to the stigma of WT, NpCIPK6-OE, and NpSnRK1-OE tobacco. The results showed that exogenous ABA could promote the germination of pollen on stigma of NpCIPK6-OE and NpSnRK1-OE tobacco (Fig. 6 a, b), and the ROS content on stigma decreased significantly (Fig. 6 a, c). Moreover, exogenous ABA also promoted the activity of antioxidant enzymes in the stigma of transgenic tobacco with NpCIPK6-OE and NpSnRK1-OE (Fig. 6 e, f, g), which may be the main reason for the decrease of ROS content.

### Knocking down *NpCIPK6* and *NpSnRK1* promotes fruit setting in intergenus hybrids of waterlily

**Figure 7:**
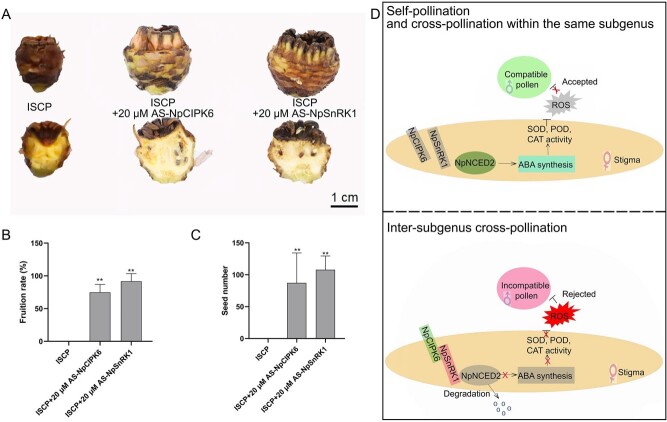
Silencing of *NpCIPK6* and *NpSnRK1* promotes fruiting in intergeneric crosses between water lily subgenera. (A) Fruits of intergeneric crosses between water lily subgenera obtained after the AS-NpCIPK6 and AS-NpSnRK1 treatments. Scale bar = 1 cm. (B) Comparison of fruiting among crosses between water lily subgenera treated with AS-NpCIPK6 or AS-NpSnRK1. (C) Comparison of number of seeds obtained from crosses between water lily subgenera treated with AS-NpCIPK6 or AS-NpSnRK1. ISCP in the figure indicates intersubgenus cross-pollinated. (D) Working model of the NpCIPK6–NpSnRK1–NpNCED2 module in regulating the formation of intersubgenus hybridization barrier in water lily. The data presented are the mean values ± SD from six independent replicates. Statistical analysis using a two-sided Student’s *t*-test revealed significant differences (*P* < 0.01) denoted by **.

We also assessed fruit setting of intersubgeneric hybridization in water lily. The fruits of *N.* “Peter Slocum” did not expand at the time of ISCP treatment and appeared to brown and rot at 25 days after pollination (Fig. 7a). We treated the stigmas of *N.* ‘Peter Slocum’ with AS-NpCIPK6 and AS-NpSnRK1, followed by intersubgeneric cross-pollination. After 25 days, we found that after AS-NpCIPK6 and AS-NpSnRK1 treatment, browning and rotting were ameliorated and fruit setting increased significantly in intersubgeneric-crossed water lily (Fig. 7a–c). Collectively, our results confirmed that the NpCIPK6-NpSnRK1 module hinders ABA synthesis on water lily stigmas by promoting protein degradation of NpNCED2 (Fig. 7d). The decrease in ABA content within water lily stigmas led to a suppression of the enzymatic activities of antioxidant enzymes, including SOD, POD, and CAT. Consequently, this inhibition facilitated the significant accumulation of ROS within the stigmas, thereby hindering the germination of incompatible pollen on the water lily stigmas.

## Discussion

The interaction between pollen and stigma is the first determinant step of cross-pollination [25–27]. In nature, plants often maintain species integrity as well as stigma health by repelling the germination of unaffiliated pollen on their stigmas and impeding harmful invaders [28, 29]. In response to compatible pollen, the stigma initiates a response that helps the pollen to germinate and grow into the ovule to complete fertilization [30, 31]. In water lily, prefertilization barriers are a major impediment to successful hybridization between water lily subgenera [1, 6, 7]. An understanding of the communication between water lily pollen and stigma is important for breaking the intersubgeneric hybridization barriers in water lily.

The germination and development of pollen on the stigma is closely associated with the regulation of hormones [12, 32]. ABA plays diverse roles in plant growth, development, and responses to abiotic stress [33] Increasing evidence suggests that ABA also participates in pollen development and pollen tube growth [11–14]. It is believed that excessive accumulation of ABA contributes to pollen sterility under conditions of cold, heat, and drought stress [14, 16, 33–35]. However, several studies have demonstrated that ABA can facilitate pollen germination and development in some plant species. For example, in tomato, ABA regulates pollen maturation by regulating the expression of specific genes involved in anther development [13, 14]. Similarly, in *Capsicum annuum*, abnormal pollen development causing fertility issues is linked to ABA degradation mediated by the cytochrome *P450* gene through hydroxylation [20]. Notably, endogenous ABA impedes pollen germination under certain abiotic stresses, including high and low temperatures [36]. In contrast, exogenous ABA has been shown to facilitate pollen germination and pollen tube elongation in self-parental and asexual lines of *Petunia hybrida* L [37]. These findings indicate that ABA is regulated differently in different species in terms of pollen fertility, depending on the dynamic equilibrium between the level of ABA and the site of ABA accumulation.

Most studies on the regulation of plant reproduction by ABA have focused on stamens, and few studies have addressed the variations of ABA levels in stigmas. In a preliminary study, we found that the change in the ABA level in stigma is one of the main causes of intersubgeneric hybridization barrier in water lily, and application of ABA can break this barrier [21]. By contrast, NpCIPK6, which is highly expressed in the ISCP-treated stigma, inhibits ABA synthesis to promote the formation of intersubgeneric hybridization barriers in water lily (Fig. 1a, d). In addition, NpCIPK6 enhances the kinase activity of NpSnRK1 (Fig. 3a), a downstream target of NpCIPK6, to suppress ABA synthesis by promoting the degradation of NpNCED2, a key enzyme of ABA biosynthesis (Figs. 3 and 4). In previous studies, CIPK6 and SnRK1, as two classical protein kinases, usually act through protein phosphorylation. For example, GhCIPK6 phosphorylates GhTST2 to regulate glucose transport from cotton to vacuoles [38]. SlCIPK6-phosphorylated SlRd2 is involved in the regulation of tomato immunity and programmed cell death by regulating ROS content changes in tomato plants [39]. SnRK1 is an important energy sensor in plants, and has been reported to interact with protein kinases, transcription factors, and some metabolite-related synthases and affect the stability of these target proteins through phosphorylation [40–44]. Members of the CIPK family have also been reported to interact with SnRK1. In rice, OsCIPK15 can regulate sugar and energy production through interaction with SnRK1A, and thus help rice cope with flooding stress [45]. AtCIPK14 has also been reported to interact with AtSnRK1.1 and AtSnRK1.2 to participate in sugar signaling in *Arabidopsis thaliana* [46]. In this study, our results also demonstrate the interaction between NpCIPK6 and NpSnRK1 and speculate that NpCIPK6 and NpSnRK1 may regulate the stability of downstream target proteins through phosphorylation modification. In summary, our data suggest that the NpCIPK6–NpSnRK1 module facilitates the formation of intersubgeneric hybridization barriers in water lily by degrading NpNCED2 to suppress ABA synthesis upon ISCP treatment.

ROS are known to play a crucial regulatory role in pollen–stigma interactions, as previously reported in multiple studies [47–49]. In angiosperms, such as *Arabidopsis* and *Glycaceae*, ROS accumulate at significantly higher levels in mature stigmas than in bud-stage stigmas [50–52] to repel incompatible pollen and microorganism invasion [53–55]. The involvement of ROS in the reproduction of plants has been demonstrated by previous studies [56–59]. For example, changes in the ROS level in the stigma have been shown to affect self-incompatibility and interspecific hybridization barriers in *Brassica* [48, 49], pollen hydration in papilla cells, and germination in *Arabidopsis* [47]. Furthermore, the occurrence of intersubgenus hybridization barriers in water lily has been linked to alterations in the level of ROS within the stigma [7, 22]. Key antioxidant enzymes such as SOD, POD, and CAT are integral in the scavenging process of ROS. These enzymes have been extensively acknowledged for their involvement in regulating the homeostasis of ROS content within the stigma and facilitating plant reproduction [23, 24]. Previously, we found that ABA application could promote ROS scavenging from the stigma, which was mediated by the increased activities of antioxidant enzymes such as SOD, POD, and CAT [21]. Consistent with our previous findings, this study shows that the knockdown of *NpCIPK6* or *NpSnRK1* in ISCP-treated stigma facilitates ROS scavenging and promotes SOD, POD, and CAT activities, which are likely caused by a high ABA content.

Signaling between the pollen and stigma promotes the growth of compatible pollen while preventing the entry of incompatible pollen and microorganisms, which serves as an important mechanism for maintaining the integrity of the species and the health of the stigma [47–49]. Our results support a model that describes the rejection of incompatible pollen by the stigma and the formation of intersubgenus hybridization barriers in water lily (Fig. 7d). In this model, NpCIPK6 is activated upon the induction of incompatible signals generated by ISCP treatment. Activated NpCIPK6 interacts with NpSnRK1 to promote its kinase activity and with NpNCED2 to promote its degradation, leading to the inhibition of ABA synthesis and the attenuation of the activation of the ABA-mediated antioxidant system. ROS accumulation in ISCP-treated stigma prohibits the germination of incompatible pollen on the stigma. Promoting ABA synthesis reduces the accumulation of ROS in the ISCP-treated stigma, thereby breaking the intersubgenus fertilization barriers in water lily and facilitating seed setting. The findings of this study increase our understanding of intersubgenus fertilization barriers in water lily, and they serve as a foundation for improving breeding efficiency and enriching the germplasm resources of water lily.

## Materials and methods

### Plant materials and growth conditions

In this study, the hardy water lily (*Nymphaea* ‘Peter Slocum’) and the tropical water lily (*Nymphaea* ‘NangKwaug Fah’) were utilized as our primary experimental materials (Fig. S1). These botanical materials are maintained within the BaiMa Education Demonstration Base of Nanjing Agricultural University. The hybridization procedures were executed in accordance with established methodologies as detailed previously [6].

### Sequence alignment and phylogenetic analysis

Sequences highly homologous to NpCIPK6 and NpSnRK1 in different species were screened using the National Center for Biotechnology Information database (https://www.ncbi.nlm.nih.gov/). Amino acid sequences from diverse species were aligned using DNAMAN 6.0 software. Subsequently, full-length amino acid sequences of CIPKs and SnRK1 proteins from different species were aligned utilizing the muscle software, employing default parameters. To infer evolutionary relationships, a neighbor-joining (NJ) phylogenetic tree was constructed using MEGA 7.0 software, with a bootstrap replication number set at 1000 [60]. Furthermore, the conserved structural domains of the genes were meticulously analyzed utilizing the MEME database (https://meme-suite.org/meme/tools/meme).

### Oligonucleotide design and treatment

Ortho- and antisense oligonucleotides (S-ODN, AS-ODN) were devised utilizing the cDNA sequences of *NpCIPK6*, *NpSnRK1*, and *NpNCED2* (The GeneBank number is visible in Table S1), employing the Sfold platform (http://sfold.wadsworth.org/cgi-bin/soligo.pl), as detailed in prior studies [48, 61]. Potential off-target effects were scrutinized through BLAST analysis (https://blast.ncbi.nlm.nih.gov/Blast.cgi). To ensure stability, three nucleotide bases at both the 5′ and 3′ termini of the S-ODN and AS-ODN were modified with sulfur. The synthesis of these oligonucleotides was entrusted to Beijing Tsingke Biotech Co., Ltd (Beijing, China). Following synthesis, the S- or AS-ODN underwent dilution in a buffer solution containing 5 mM CaCl_2_, 5 mM KCl, 0.01% H_3_BO_3_, 1 mM MgSO_4_·7H_2_O, 10% sucrose, 0.8% agarose, adjusted to pH 7.5 [48]. Subsequently, the diluted S- or AS-ODN was placed on the water lily stigma for treatment. The S- or As-ODN sequences used in this experiment can be seen in Table S3.

Prior to ODN treatment, stamens were meticulously excised from the flowers of chosen water lily plants. Subsequently, the designated ODN was administered to the defolliculated stigma of the water lily at a concentration of 20 μM. After ODN treatment for 1 h, the stigma of *N.* ‘Peter Slocum’ was pollinated with pollen from *N.* ‘NangKwaug Fah’.

### Pollen tube visualization analysis

Six hours after pollination, the pollinated stigmas of *N.* ‘Peter Slocum’ were collected, fixed in formaldehyde-acetic acid-ethanol fixative (70%) for 6 h, and placed in 1 M NaOH overnight for softening. The softened stigmas were stained using aniline blue as described previously [53]. Preliminary slides were prepared, and the columns were observed at 405 nm with a Nikon model fluorescence microscope (Tokyo, Japan) and photographed with a Nikon DS-Ri2 digital camera.

### Determination of ABA content

The quantification of ABA content in water lily stigma followed established protocols [62]. Briefly, 0.1 g sample of stigma was weighed, promptly frozen in liquid nitrogen, and finely ground into powder. Subsequently, the ABA extraction was performed using an acetone:water:acetic acid (80:19:1, v:v:v) solution. The 0.0025 g ABA-d6 was added to the 2.5 ml extraction solution as the internal standard. After extraction, 1 ml of the sample was separated by gravity flow on an Oasis HLB column (Waters, Milford, MA, USA). The eluted metabolites were then dried and resuspended in a solution composed of water:acetic acid:formic acid (94.9:5:0.1, v:v:v). The resulting mixture was subjected to liquid chromatography-mass spectrometry/mass spectrometry analysis for metabolite profiling. Three stigmas from the same water lily plant were used as three biological replicates.

### RNA, DNA extraction and quantitative real-time PCR

Total RNA was extracted utilizing the FastPure Universal Plant Total RNA Isolation Kit (Vazyme, Nanjing, China) in accordance with the manufacturer’s guidelines. The tobacco DNA was isolated using the Plant Genomic DNA Kit (Tiangen, Beijing, China). The integrity and concentration of both total RNA and genomic DNA were assessed through 1% agarose gel electrophoresis and NanoDrop 1000 spectroscopy (Thermo Fisher Scientific, Waltham, MA, USA). First-strand cDNA synthesis was performed utilizing the HiScript III RT SuperMix for qPCR (gDNA wiper) Kit (Vazyme, Nanjing, China), with 1 μg of total RNA employed in each 20-μl reverse transcription reaction. Subsequently, the cDNA products were diluted 10-fold with deionized water prior to utilization. qRT-PCR assays were performed utilizing the ChamQ SYBR qPCR Master Mix Kit (Vazyme, Nanjing, China) with a CFX96 Touch Real Time PCR Detection System (BioRad, Hercules, CA, USA). Each reaction comprised 2 μl of cDNA, and experimental setup and conditions adhered to the manufacturer’s guidelines. Primer sequences for qRT-PCR were custom-designed using DNAMAN 6 software (Table S2). The water lily *Actin11* gene was employed as an internal reference ^63^, while the *NtActin* gene in tobacco was used to identify transgenic tobacco [64]. Each treatment was subjected to three biological and three technical replicates. Gene expression levels were determined utilizing the 2^-ΔΔCt^ method [65, 66].

### SnRK1 enzyme activity assay

The kinase activity of SnRK1 in the stigma of water lily was detected using the plant Snf1 associated protein kinase (SnRK1) enzymic immunoassay kit (Jonln, JL49688). The specific experimental procedures, refer to the kit instructions.

### Vector construction and plant transformation

The complete coding sequences (CDS) of *NpCIPK6*, *NpSnRK1*, and *NpNCED2* were integrated into *pCambia1300-35S: GFP* vectors, and the full-length CDS of *NpSnRK1* and *NpNCED2* were incorporated into *pBI121-GUS* vectors through homologous recombination, following the protocols outlined in the CloneExpress II One Step Cloning Kit (Vazyme, Nanjing, China). Details of the primers utilized for vector construction are provided in ‘Table S2’. Subsequently, the engineered plasmids were introduced into *Agrobacterium rhizogenes* GV3101 strain and subsequently utilized for infiltrating *N. benthamiana* leaves.

### Subcellular localization of NpSnRK1 and NpCIPK6

The *Agrobacterium tumefaciens* GV3101 strain carrying *pCambia1300-35S: NpCIPK6-GFP* or *pCambia1300-35S: NpSnRK1-GFP* was infiltrated into *N. benthamiana* leaves to determine the subcellular localization of NpSnRK1 and NpCIPK6. Green fluorescent protein fluorescence was observed 48 h after infiltration using a Zeiss LSM 800 confocal laser scanning microscope (Carl Zeiss Microscopy, White Plains, NY, USA).

### Yeast two-hybrid assay

The full-length CDS sequences of *NpCIPK6* and *NpSnRK1* were separately incorporated into the *pGBKT7* vector. Similarly, the CDS of *NpSnRK1*, *NpNCED1*, *NpNCED2*, and *NpNCED4* were individually cloned into the *pGADT7* vector. Subsequently, the resulting recombinant *pGBKT7* plasmids were transfected into yeast cells, and the self-activation of *NpCIPK6-BD* and *NpSnRK1*-BD was evaluated on selective medium devoid of -Trp and -Leu. For the analysis of protein–protein interactions, the recombinant *pGADT7* and *pGBKT7* plasmids were introduced into yeast cells, which were then cultured on selective medium lacking -Trp, -Leu, -His, and -Ade.

### Luciferase complementation assay

The full-length CDS sequences of *NpCIPK6*, *NpSnRK1*, and *NpNCED2* were amplified by RT-PCR, and subsequently cloned into the vectors *pCAMBIA1300-nLUC* and *pCAMBIA1300-cLUC* to produce *NpCIPK6-nLUC*, *NpSnRK1-cLUC*, and *NpNCED2-nLUC* constructs, respectively. Following construction, these constructs were separately introduced into *N. benthamiana* leaves through *Agrobacterium*-mediated transformation. Leaves of *N. benthamiana* coexpressing *nLUC* and *cLUC* served as a control. Luciferase activity was quantified utilizing the Berthold LB985 Plant Live Imaging System (Stuttgart, Germany).

### Pull-down assay

The full-length CDS sequences of *NpCIPK6* and *NpNCED2* were individually incorporated into the *pET-32a* vector, featuring a His tag, while the full-length CDS sequence of *NpSnRK1* was integrated into the *pGEX-4 T-1* vector, which includes a GST tag. These recombinant plasmids were introduced into *Escherichia coli* BL21 cells to facilitate the expression of *NpCIPK6-His*, *NpNCED2-His*, and *NpSnRK1-GST* fusion proteins. Following expression, equivalent quantities of the fusion proteins with different tags were combined and then allowed to incubate at 4°C for 12 h. Subsequently, a pull-down assay was conducted utilizing the His-Tagged Protein Purification Kit (Soluble Protein) (Cowin Biotech, Beijing, China).

### Protoplast isolation and bimolecular fluorescence complementation assay

*Nicotiana benthamiana* protoplasts were isolated following a previously established protocol with slight modifications [67]. For protoplast extraction, perforated *N. benthamiana* leaves were placed in extraction buffer (3.05 g/l Gamborg B5 salt medium, 500 mg/l MES, 750 mg/l CaCl_2_·2H_2_O, 250 mg/l NH_4_NO_3,_ pH 5.7, 0.2% [w/v] macerozyme, 0.4% [w/v] cellulase) and incubated at 25°C overnight in the dark. The protoplasts were washed three times with wash buffer (137 g/l sucrose, 2.4 g/l HEPES, 6 g/l KCl, 600 mg/l CaCl_2_·2H_2_O, pH 7.2) and resuspended in the wash buffer.

For the BiFC assay, the full-length CDS sequences of *NpCIPK6* and *NpNCED2* were cloned into *pCambia1300-nYFP* vector, and the full-length CDS sequences of *NpSnRK1* were cloned into *pCambia1300-cYFP* vector, through homologous recombination. A 600-μl aliquot of extracted protoplasts was combined with 2–6 mg of recombinant plasmid containing the *NpCIPK6*, *NpNCED2*, or *NpSnRK1* sequence using the Xcell Gene Pulse Generator (BioRad), which applied a 160-V pulse for 10 ms as previously described [68]. After transfection, 2 ml of wash buffer, which was used for the resuspension of protoplasts, was added to each sample, and yellow fluorescent protein was observed using a Zeiss LSM 800 confocal laser scanning microscope (Carl Zeiss Microscopy, White Plains, NY, USA) after 16–24 h at 25°C in the dark.

### Protein extraction and western blot analysis

Plant proteins were extracted using a method previously outlined [69], with certain modifications applied. Briefly, total proteins were extracted from frozen leaf materials utilizing extraction buffer consisting of 56 mM Na_2_CO_3_, 56 mM DTT, 2% SDS (w/v), 12% sucrose (w/v), and 2 M EDTA. A 0.1 g sample powder was suspended in 500 μl of extraction buffer, followed by an incubation period of 10 min at 90°C and subsequent centrifugation at 16 000 g for 10 min at room temperature. The extracted proteins were then separated on 12% polyacrylamide SDS gels and transferred onto polyvinylpyrrolidone membranes.

These membranes were then blocked for 1 h in PBS-T buffer (50 mM Tris–HCl, 150 mM NaCl, pH 7.5; 0.1% Tween-20) containing 4% milk (w/v). Following blocking, the membranes were incubated overnight at 4°C in a 1% milk solution containing the primary antibody (anti-GFP or anti-Actin, 1:2000, sourced from Shanghai, China). Subsequently, the membranes were washed and exposed to the secondary antibody (HRP-conjugated goat anti-mouse IgG, 1:10 000 in 1% milk–PBS-T) for 2 h at 4°C. Immunoreactive proteins were detected using the Clarity Western ECL Substrate Kit (BioRad) and captured using the ChemiDoc MP System (BioRad).

### GUS assay

The procedure for GUS staining involved immersing the infiltrated *N. benthamiana* leaves into a GUS staining buffer containing 0.5 μl 1 × Gluc, 0.075 M sodium phosphate (pH 7.2), 0.05 mM K_4_[Fe(CN)]_6_^**.**^3(H_2_O), 0.05 mM K_3_[Fe(CN)_6_], 10 mM EDTA, 20% [v/v] methanol, and 0.1% [v/v] Triton X-100. Subsequently, the leaves were incubated overnight at 37°C.

For the GUS activity assay, 500 mg of *N. benthamiana* leaves, infected for 48 h, were ground using liquid nitrogen. GUS proteins were extracted by adding 1 ml of GUS extraction buffer composed of 50 mM sodium phosphate (pH 7.0), 10 mM EDTA, 0.1% [v/v] Triton X-100, and 0.1% [v/v] n-lauroylsarcosine. The GUS activity assay, as outlined in previous studies [70, 71], was conducted with each assay being replicated independently at least three times.

### Detection of reactive oxygen species

The ROS content was determined as previously described [48]. Six hours after pollination, the stigma of water lily was soaked in MES-KCl buffer (10 mM MES, 5 mM KCl, 50 mM CaCl_2_, pH 6.15) for 30 min. After staining with 50 μM 2′,7′-dichlorodihydrofluorescein diacetate (H_2_DCFDA) for 1–2 h, the column was washed at least three times with PBS. A preliminary slide of the stigma was prepared for observation of enhanced green fluorescent protein (Ex470–440, DM4951p, BA525/550). The fluorescence intensity of ROS was quantified using Image J software (National Institutes of Health, Bethesda, MD, USA).

### Antioxidant enzyme activity assay

For the assessment of antioxidant enzyme activity, total proteins were extracted from ~0.1 g of water lily stigma using 1 ml ice-cold extraction buffer composed of 25 mM HEPES buffer (pH 7.8), 0.2 mM EDTA, 2 mM ascorbic acid, and 2% [v/v] polyvinylpyrrolidone. The resulting homogenate underwent centrifugation at 12 000 × g for 20 min at 4°C, and the supernatant was collected for subsequent enzyme activity assays. The activities of SOD, POD, and CAT were determined following established protocols [72].

### Tobacco transformation

The leaf disk method was used to carry out transgenic operation of tobacco (*N. tabacum* L.) [64]. *pSak277-35S: NpCIPK6* and *pSak277-35S: NpSnRK1* were transferred into tobacco by *Agrobacterium*-mediated method.

### Statistical analysis

Intergroup significant differences were assessed utilizing one-way analysis of variance (ANOVA), and *post hoc* pairwise comparisons were conducted employing Tukey’s test, with significance set at *P* < 0.05. Two-group comparisons were executed utilizing a two-tailed Student’s *t*-test. The statistical analyses were carried out using SPSS version 28.0.1.1 software.

## Supplementary Material

Web_Material_uhae289
