# Supplementary material for: NpCIPK6–NpSnRK1 module facilitates intersubgeneric hybridization barriers in water lily (Nymphaea) by reducing abscisic acid content
Source: Hortic Res. 2024 Oct 23;12(1):uhae289. doi: 10.1093/hr/uhae289 (PMC11775591; doi:10.1093/hr/uhae289)
Supplement: Web_Material_uhae289 [file web_material_uhae289.zip › Supplementary Pictures.docx]

| **Supporting Information legends:**  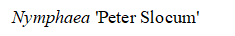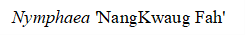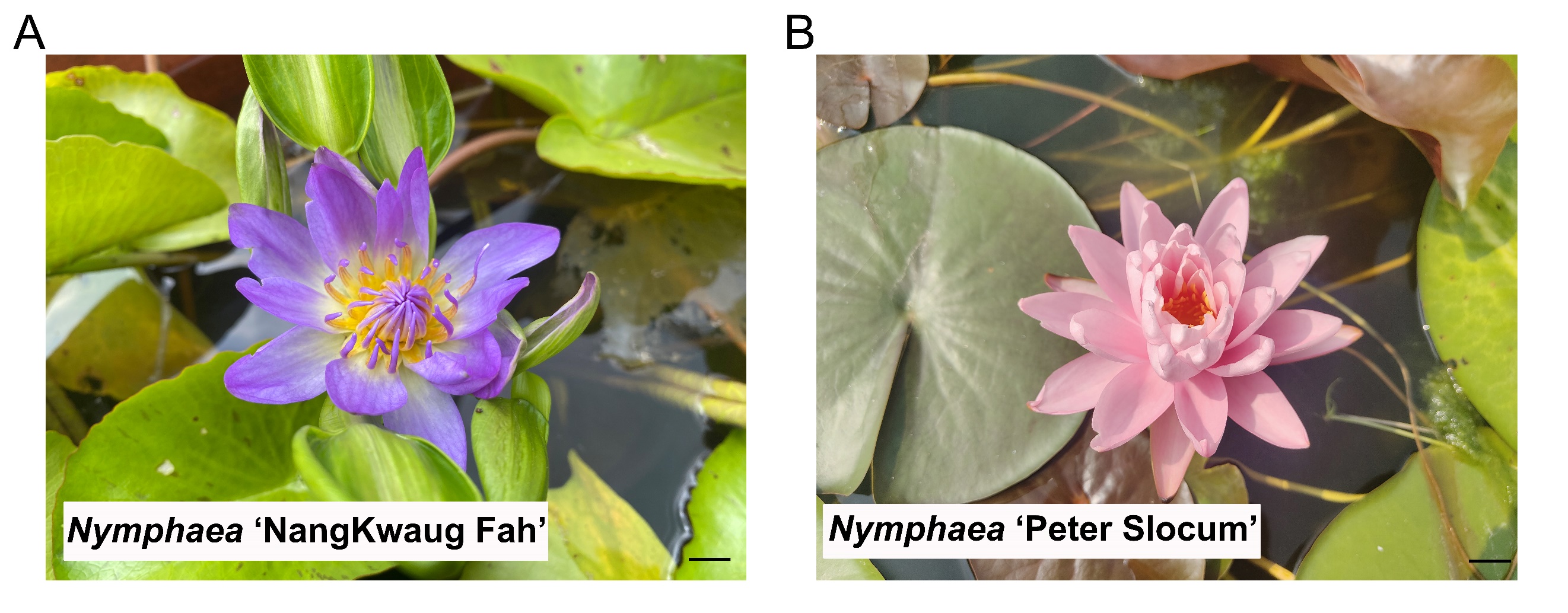 |
| --- |
| **Figure S1.** The water lily plants used in this study. (A) Water lily plants used for parental use. (B) Waterlily plants for maternal use. Scale bar = 1 cm. |
| 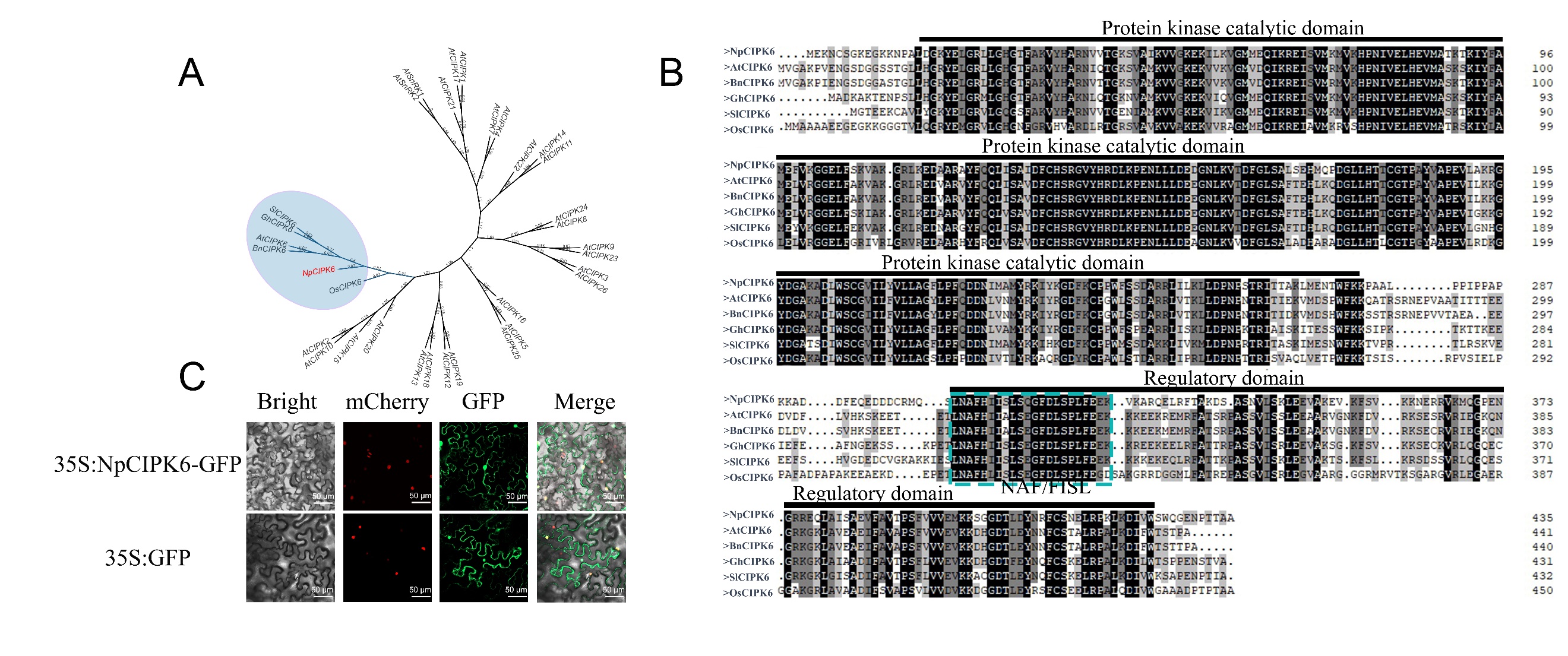  **Figure S2.** Phylogenetic analysis, multiple sequence alignment, and subcellular localization of CBL-interacting protein kinase 6 (CIPK6). (A) Phylogenetic tree of NpCIPK6 with CIPKs from *Arabidopsis*, *Oryza sativa*, *Solanum lycopersicum*, *Glycine max* and *Hordeum vulgare*. Bootstraps values with 1000 replicates are shown on branches. (B) Alignment of the full protein sequences of NpCIPK6 and its homologs in water lily and other species. Conserved residues are marked with black boxes, and NAF/FISE structural domains are marked with blue dashed lines. Comparisons were generated using DNAMAN 6.0 software. (C) Localization of NpCIPK6-GFP in *N. benthamiana* leaves. Scale bar = 50 μm.  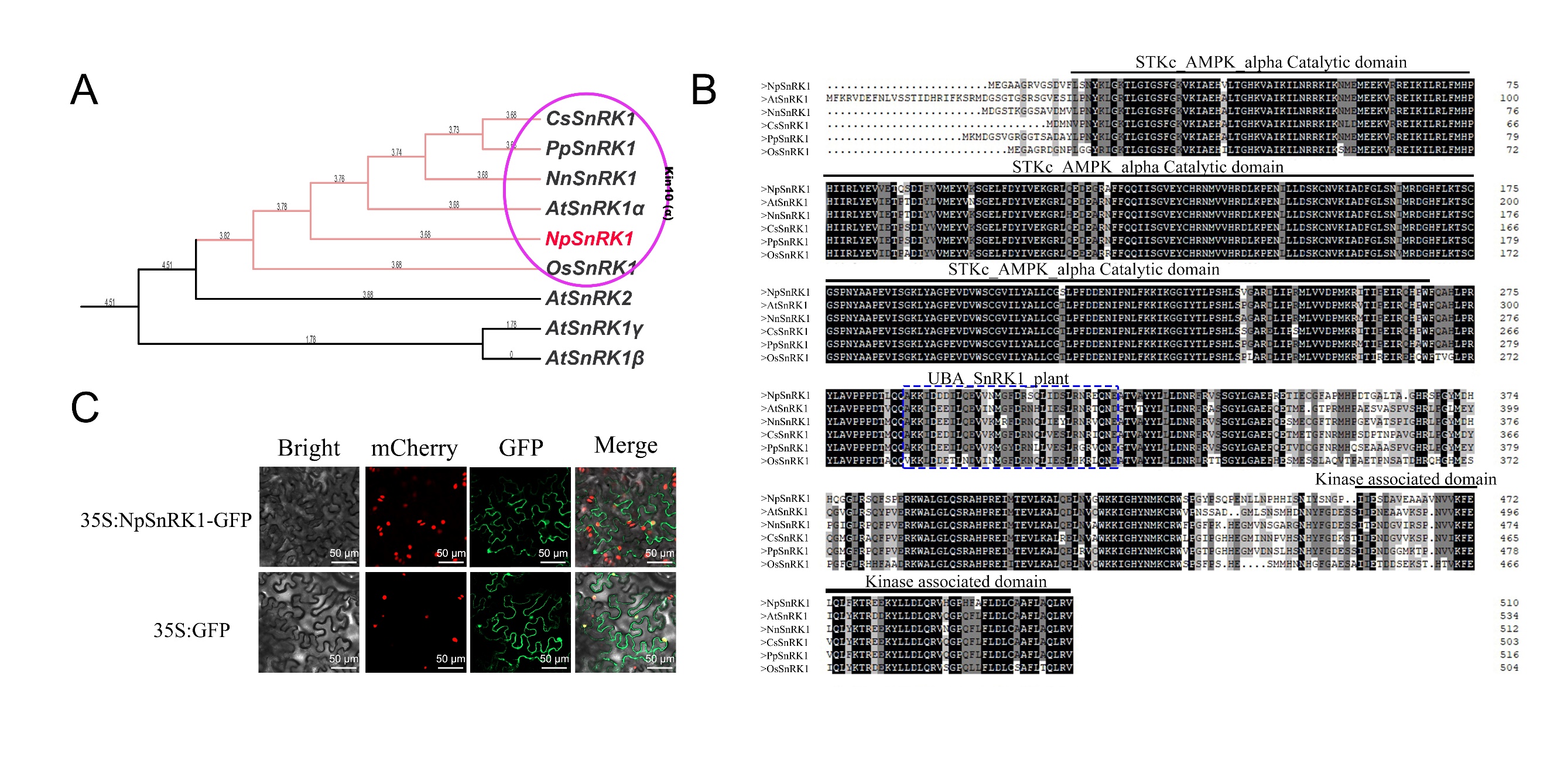 |
| **Figure S3.** Phylogenetic, sequence alignment and subcellular localization analysis of SnRK1. (A) Phylogenetic relationship of NpSnRK1 with representative SnRKs from different plant species. Percentage of bootstraps with 1000 replicates is shown on the branch. (B) Alignment of the full amino acid sequences of NpSnRK1 homologs in water lily and other species. Conserved residues are marked with black boxes, and UBA-SnRK1 plant structural domains are marked with blue dashed lines. Comparisons were generated using DNAMAN software. (C) Localization of NpSnRK1-GFP in *N. benthamiana*. Scale bar = 50 μm. |
| 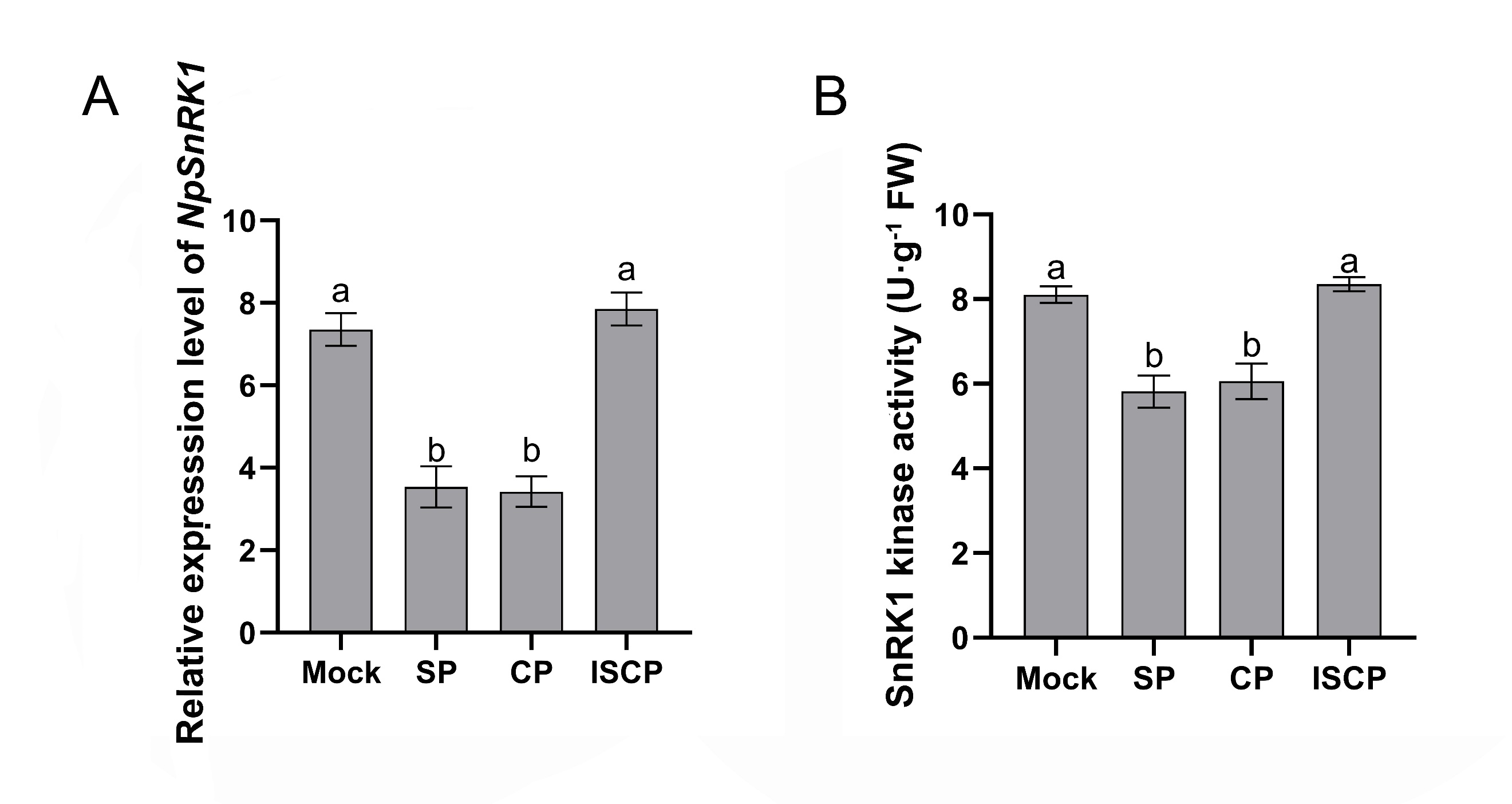 |
| **Figure S4.** Response of NpSnRK1 to different pollination combinations of water lily treatments. The expression (A) and kinase activity (B) of SnRK1 in stigmas was analyzed for different pollination treatments, including unpollinated (Mock), self-pollinated (SP), cross-pollinated within the same subgenus (CP), and inter-subgenus cross-pollinated (ISCP). The data presented are the mean values ± standard deviation (SD) from three independent replicates. Statistical analysis using one-way ANOVA revealed significant differences among the treatments (*P* < 0.05), as indicated by different letters. |
| 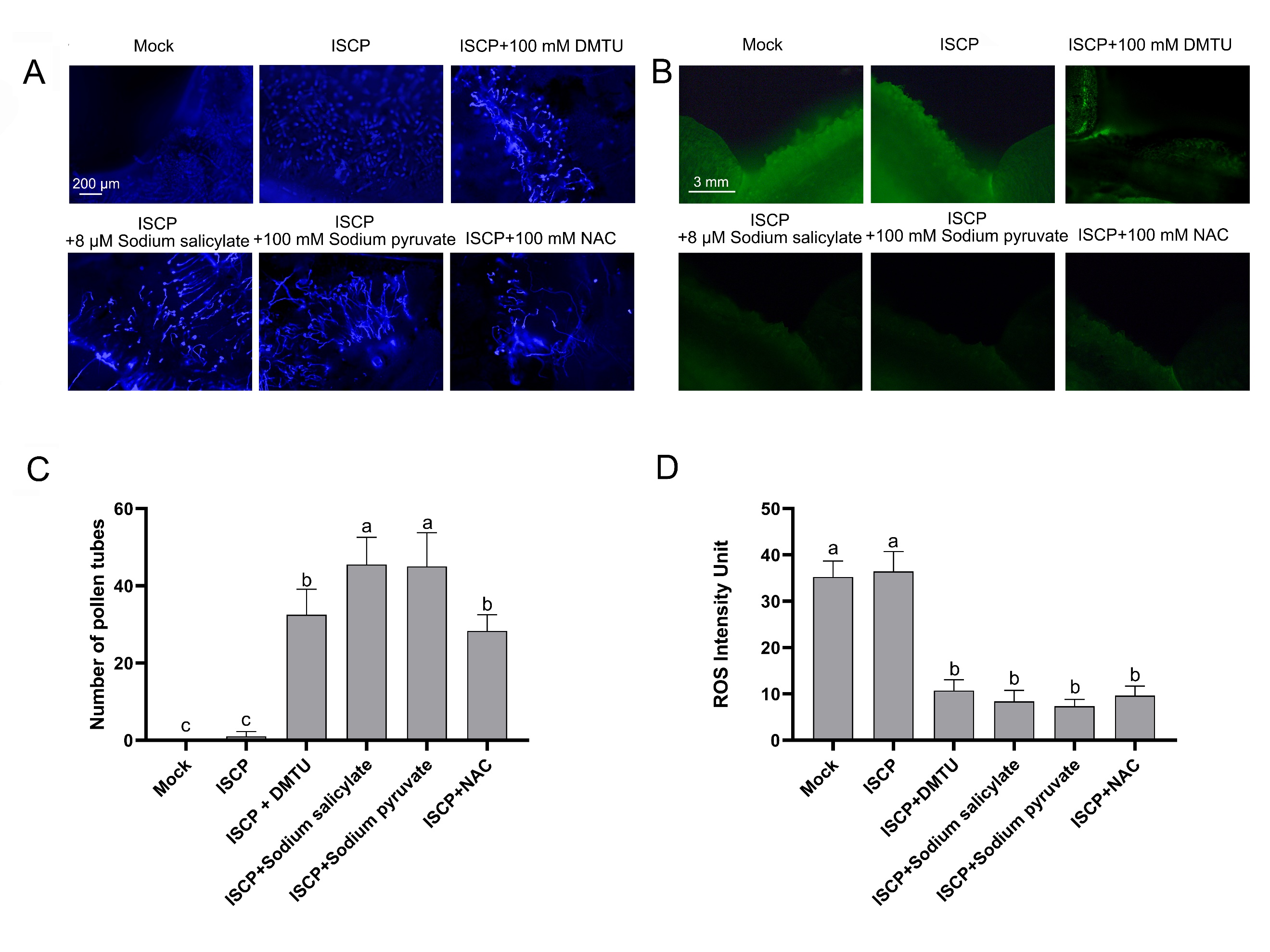 |
| **Figure S5.** The high content of ROS is the key factor leading to the inter-genus hybridization barrier of water lily. (A) The growth of inter-genus hybrid pollen on ISCP stigma. (B) Statistics of pollen germination numbers. (C) Statistics of pollen germination numbers. (D) Changes of ROS content in the stigma of water lily. The data presented are the mean values ± standard deviation (SD) from three independent replicates. Statistical analysis using a two-sided Student's *t*-test revealed significant differences (*P* < 0.01) denoted by **. |
| 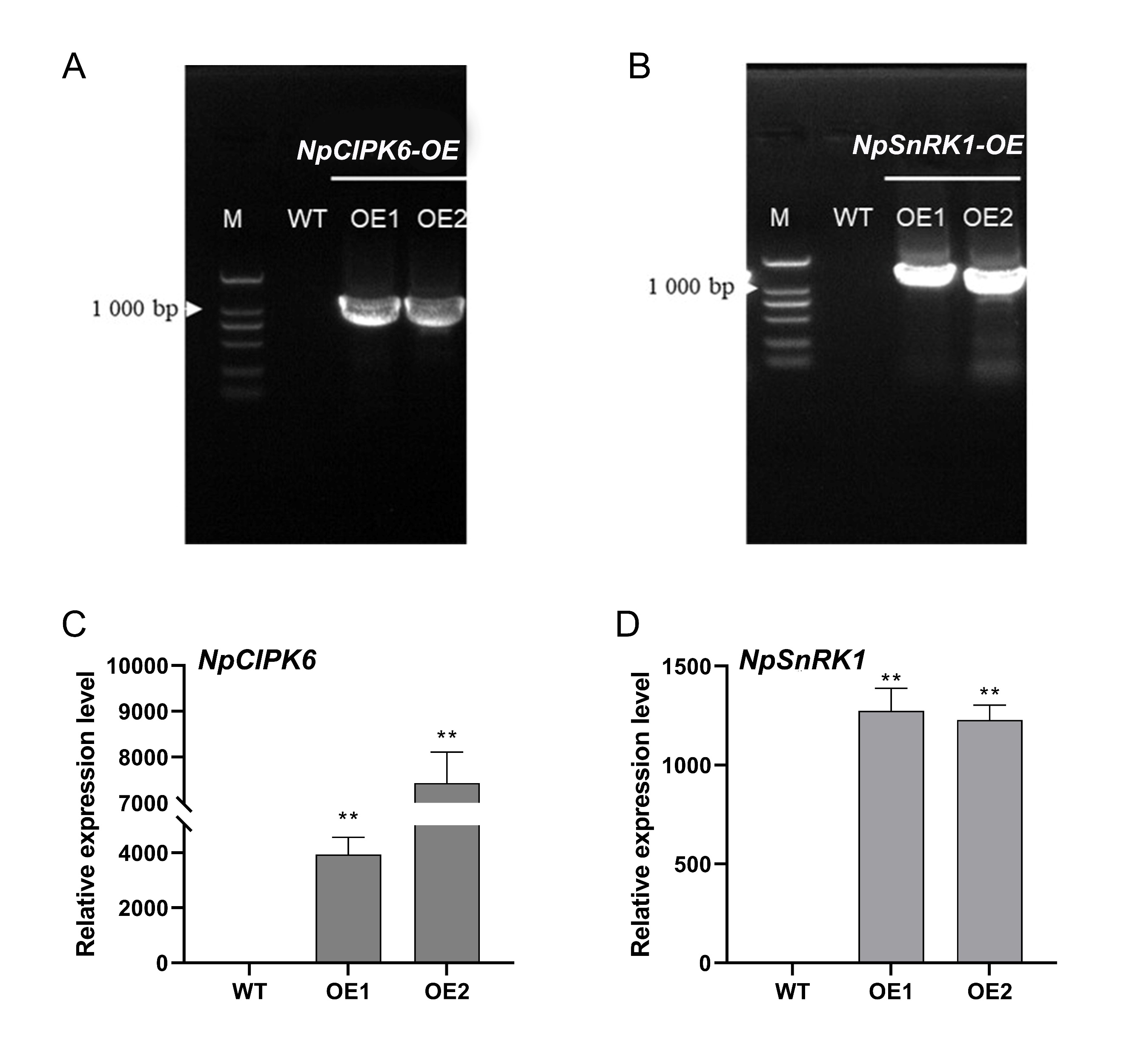 |
| **Figure S6.** Identification of transgenic tobacco plants. (A) DNA level identification of NpCIPK6 transgenic tobacco; (B) DNA level identification of NpSnRK1 transgenic tobacco; (C) qRT-PCR analysis of *NpCIPK6* expression in wild type and transgenic tobacco; (D) qRT-PCR was used to analyze the expression of *NpSnRK1* in wild type and transgenic tobacco. The data presented are the mean values ± standard deviation (SD) from three independent replicates. Statistical analysis using a two-sided Student's *t*-test revealed significant differences (*P* < 0.01) denoted by **. |
